# proABC-2: PRediction of AntiBody contacts v2 and its application to information-driven docking

**DOI:** 10.1093/bioinformatics/btaa644

**Published:** 2020-07-19

**Authors:** Francesco Ambrosetti, Tobias Hegelund Olsen, Pier Paolo Olimpieri, Brian Jiménez-García, Edoardo Milanetti, Paolo Marcatilli, Alexandre M J J Bonvin

**Affiliations:** Department of Physics, Sapienza University, 00184 Rome, Italy; Department of Chemistry, Faculty of Science, Computational Structural Biology Group, Bijvoet Centre for Biomolecular Research, Utrecht University, Utrecht 3584CH, The Netherlands; Department of Health Technology, Technical University of Denmark, Kgs. Lyngby 2800, Denmark; Department of Physics, Sapienza University, 00184 Rome, Italy; Department of Chemistry, Faculty of Science, Computational Structural Biology Group, Bijvoet Centre for Biomolecular Research, Utrecht University, Utrecht 3584CH, The Netherlands; Department of Physics, Sapienza University, 00184 Rome, Italy; Fondazione Istituto Italiano di Tecnologia (IIT), Center for Life Nano Science, 00161 Rome, Italy; Department of Health Technology, Technical University of Denmark, Kgs. Lyngby 2800, Denmark; Department of Chemistry, Faculty of Science, Computational Structural Biology Group, Bijvoet Centre for Biomolecular Research, Utrecht University, Utrecht 3584CH, The Netherlands

## Abstract

**Motivation:**

Monoclonal antibodies are essential tools in the contemporary therapeutic armory. Understanding how these recognize their antigen is a fundamental step in their rational design and engineering. The rising amount of publicly available data is catalyzing the development of computational approaches able to offer valuable, faster and cheaper alternatives to classical experimental methodologies used for the study of antibody–antigen complexes.

**Results:**

Here, we present proABC-2, an update of the original random-forest antibody paratope predictor, based on a convolutional neural network algorithm. We also demonstrate how the predictions can be fruitfully used to drive the docking in HADDOCK.

**Availability and implementation:**

The proABC-2 server is freely available at: https://wenmr.science.uu.nl/proabc2/.

**Supplementary information:**

Supplementary data are available at *Bioinformatics* online.

## 1 Introduction

Monoclonal antibodies (mAbs) are now well established in the contemporary therapeutic repertoire. Indeed in 2018 12 antibodies were granted first approval by either the European Medicines Agency or by the Food and Drug Administration while about 570 are undergoing clinical development at various stages (Kaplon and Reichert, 2019). The reasons behind the increasingly consolidated use of mAbs as therapeutics should be sought in their high affinity and specificity toward their cognate antigen and their modular architecture which facilitates their engineering (Chames *et al.*, 2009). Understanding the fundamentals of antibody–antigen interactions is a critical step for the rational design and engineering of immunoglobulins. Since classical experimental approaches used to characterize antibodies (e.g. NMR, X-ray and mass spectrometry) are often expensive and time consuming, computational tools offer valuable and complementary approaches which can provide information at different levels (sequence and/or structural) (Norman *et al.*, 2019).

To this end, we previously reported a method named proABC (Olimpieri *et al.*, 2013) that can predict antibody residues forming intermolecular contacts with the cognate antigen, as well as the nature of their contacts, distinguishing between hydrogen bonds and hydrophobic interactions. proABC is based on a random forest algorithm, using the antibody heavy and light chain sequences, the hypervariable loop canonical structures and lengths (Chothia and Lesk, 1987) and the germline family as features (Schatz and Swanson, 2011). Its performance has been validated by us (Olimpieri *et al.*, 2013) and others (Peng *et al.*, 2014) demonstrating good accuracy and reliability.

Here we present proABC-2, an update of the original algorithm using the same set of features but based on a deep learning framework shown to be successful in achieving similar goals (Deac *et al.*, 2019; Liberis *et al.*, 2018). Furthermore, we show how the proABC-2 predictions can be used to drive the modeling of antibody–antigen complexes using the information-driven docking approach HADDOCK (Van Zundert *et al.*, 2016), which was recently demonstrated to be the best option of the compared methods for antibody–antigen modeling (Ambrosetti *et al.*, 2020). The method is integrated in a freely available web server that predicts paratope residues forming general contacts as well as those involved in hydrogen bonds and hydrophobic interactions.

## 2 Results

The prediction performance of proABC-2 was measured, after a 10-fold-nested cross-validation, in terms of AUC, MCC and F-score values for all the general interactions of the paratope (*Pt*), hydrophobic interactions (*Hy*) and for hydrogen bonds (*Hb*) (see Supplementary Materials). The highest performance is obtained for *Pt* (0.96, 0.57 and 0.59, respectively, for AUC, MCC and F-score) and decreases for *Hy* (0.95, 0.44 and 0.41) and *Hb* (0.94, 0.33 and 0.27). This is due to the smaller number of *Hb* and *Hy* interactions in the training set compared to the general (*Pt*) ones. When trained on the same data and in a similar approach, proABC-2 outperforms Parapred (Liberis *et al.*, 2018), one of the currently best available methods for paratope prediction. Details about the model evaluation and the comparison with Parapred are provided in the Supplementary Materials.

### 2.1 Prediction-driven docking accuracy

We investigated whether the predictions obtained from proABC-2 can be used to drive antibody–antigen docking using the HADDOCK 2.2 webserver (Van Zundert *et al.*, 2016). For unbiased predictions, the model was trained excluding all sequences sharing ≥95% sequence identity with any structure used for docking. Only residues predicted as *Pt* were used for docking (using a 0.40 cutoff). The results were compared to a previous study performed using the hypervariable loops (Ambrosetti *et al.*, 2020, Supplementary Figs S2 and S3). The performance was evaluated in terms of success rate defined as the number of complexes for which at least one acceptable, medium or high-quality complex was found in the top 1, 5, 10, 20, 50 and 100 ranked models. Supplementary Figure S2 shows the results of the docking obtained by providing to the algorithm all solvent accessible residues of the antigen and either the antibody hypervariable loops (HV-Surf) or the proABC-2 predictions (*Pt*) (Pred-Surf). The HV-Surf docking led to slightly better results for the top 1, 5 and 10 with 25.0%, 31.2% and 31.2% success rates, respectively, compared to 18.7%, 25.0% and 25.0% for Pred-Surf. The proABC-2 predictions give better results for the top 50 and 100 (50% and 62.5% success rates, respectively). Thus, even if HADDOCK is able to generate correct models, the scoring is not able to rank them in the top. As for the quality of the docking models, using the HV loop leads to better-quality models overall.

Supplementary Figure S3 shows the results of the docking obtained by providing to the algorithm a loose definition of the epitope following the definition given in Ambrosetti *et al.* (2020). In this scenario, the proABC-2 predictions led to a remarkable improvement of the Top1 success rate from 43.8% (using HV) to 62.5%. In general, the use of the proABC-2 predictions resulted in an improvement of the quality of the generated models, mainly reflected in the number of medium-quality ones. Details about the docking scenarios and settings are provided in the Supplementary Materials.

### 2.2 Web server

proABC-2 is freely available as a web server at https://wenmr.science.uu.nl/proabc2. It only requires the sequences of the heavy and light chains. The input is processed to calculate all of the sequence-derived features (germline, canonical structures and length of the HV loops), and these are passed to the CNN to make the predictions. The computation only takes a few seconds. The results page reports in a bar plot the residue probabilities of making a general, H-bond and hydrophobic interactions (see Supplementary Fig. S4). Two files (for the heavy and light chains) are provided as output, containing for each residue the different probabilities.

## 3 Conclusions

proABC-2 is based on a deep learning framework and shows a high performance with an AUC of 0.96 and an MCC of 0.57. Its predictions should be useful for antibody design such as *in silico* affinity maturation or humanization. We also demonstrated how these predictions can guide molecular docking, showing in particular that if a loose definition of the epitope region is provided, the proABC-2 predictions leads to improvements of both success rate and quality of the docked models. This suggests that different strategies might be followed depending on the available information about the epitope.

To our knowledge, proABC-2 is the only available method, specifically designed for antibodies, able to predict the paratope residues along with the type of interaction. The method is freely available as a web server and provides a straightforward user-friendly interface.

## Funding

This work was supported by the European Union Horizon 2020 BioExcel [823830] and EOSC-Hub [777536] projects.

*Conflict of Interest*: none declared.

## Supplementary Material

btaa644_Supplementary_Data
