# Supplementary material for: proABC-2: PRediction of AntiBody contacts v2 and its application to information-driven docking
Source: Bioinformatics. 2020 Jul 19;36(20):5107–8. doi: 10.1093/bioinformatics/btaa644 (PMC7755408; doi:10.1093/bioinformatics/btaa644)
Supplement: btaa644_Supplementary_Data [file btaa644_supplementary_data.pdf]

Supplementary material

proABC-2: PRediction Of AntiBody Contacts v2 and its application to information-driven docking

F. Ambrosetti[a,b,+], T. H. Olsen[c,+], P. P. Olimpieri[a], B. Jiménez-García[b], E. Milanetti[a,d], P. Marcatili[c], A.M.J.J. Bonvin[b*]

[+]authors contributed equally

*To whom correspondence should be addressed.

[a]*Department of Physics, Sapienza University, Piazzale Aldo Moro 5, 00184, Rome, Italy,*

[b]*Faculty of Science - Chemistry, Computational Structural Biology Group, Bijvoet Centre for Biomolecular Research, Utrecht University, Utrecht, The Netherlands,*

[c]*Department of Health Technology, Technical University of Denmark, Kgs. Lyngby, Denmark.*

[d]*Center for Life Nano Science@Sapienza, Istituto Italiano di Tecnologia, Viale Regina Elena 291, 00161 Rome, Italy*

Contact: a.m.j.j.bonvin@uu.nl

Dataset

The full protein data bank (PDB) was scanned using in-house Hidden Markov Models (HMM) in order to identify all the antibody structures deposited. Immunoglobulins having only one chain (nanobodies), a resolution higher than 3Å or not solved with an antigen were excluded. Finally, using *cd-hit* (Fu *et al.*, 2012) all the antibodies sharing a sequence identity higher than 95% with any other immunoglobulin of the dataset were removed.
We ended up with a dataset of 769 complexes (*CNN-dataset*) which was used to train the model.
For the docking studies a dataset of 16 complexes (*Docking-set*), all with available unbound structures, corresponding to the new antibody-antigen entries of the protein-protein benchmark version 5.0 was used (Vreven *et al.*, 2015).
Moreover, for a fair comparison with Parapred, the same structures used to train it (*Parapred-set*) have been used to train proABC-2.

Interaction calculation

For all the complexes of the CNN-dataset, the non-covalent interactions including intermolecular hydrogen bonds and hydrophobic interactions were calculated. Non-covalent interactions were determined using a distance cut-off of 3.9Å. Hydrogen bonds were calculated by defining donors (D) as any N/O/F/S connected to a hydrogen atom and acceptors (A) as any N/O/F/S within a distance threshold (2.5Å) of that hydrogen and by filtering the matches for D-H-A triplets with a minimum angle of 120 degrees (Baker and Hubbard, 1984). Finally, hydrophobic interactions were computed using a distance cut-off of 4.4Å between any heavy atom of two hydrophobic residues (Bissantz *et al.*, 2010).

General contacts were calculated using an in-house R script while H-bond and hydrophobic interactions were determined using *interfacea* (Rodrigues *et al.*, 2019).

Neural network features

In order to train the CNN a specific set of features was used:

1. *Light and heavy chain sequences* aligned using HMM profiles. In particular for the H3 alignment insertions were positioned in the middle between the two conserved residues Cys92 and Gly104 according to the previously described method (Morea *et al.*, 1998). Each sequence position was considered as a variable. To allow the textual information of a sequence to be processed by an algorithm, each residue was converted into numerical values using one-hot encoding, the representation of categorical variables (i.e. a residue) as binary vectors. Here, a 20x1 vector has been used consisting of all zeros except at the index of the given residue, which was marked with a 1. Concurrent, a 20x1 vector of only zeros represented a gap. The heavy and light chains were represented by a 297x20 array.

2. *Hypervariable loops canonical structures* calculated according to the key residues found within and outside the loops (Chothia and Lesk, 1987; Vargas-Madrazo and Paz-García, 2002; Morea *et al.*, 1998). One-hot encoding was used.

3. *Length of the hypervariable loops* defined according to the Chothia numbering scheme.

4. *Germline family* and *source organism* determined using *igblastp* (Ye *et al.*, 2013). One-hot encoding was used.

Convolutional Neural Network (CNN)

The neural network used by proABC-2 consists of three convolutional modules (Conv11, Conv12 and Conv2), a fully connected feed-forward module (Ff1) and an output layer (Figure S1). Conv11 and Conv12 are identical and consist of three parts; a 1D convolutional layer with 32 filters of size 3x1 and a stride of 1, followed by a 1D max pooling layer of size 10x1 and a stride of 3 and finally a dropout layer with a dropout rate of 0.15. Conv2 also consists of three parts; a 1D convolutional layer with 64 filters of size 3x1 and a stride of 1, followed by a 1D average pooling layer of size 6x1 and a stride of 3 and finally a dropout layer with a dropout rate of 0.15. Ff1 consists of a fully connected layer with 512 nodes followed by a dropout layer with a dropout rate of 0.10. The final output layer has for each of the 297 residues 3 nodes, predicting the general interactions, H-bonds and hydrophobic interactions, amounting to 891 nodes. The model was constructed using the python package *Tensorflow* (Abadi *et al.*, 2016).
These modules are combined in the following way. The one-hot encoded heavy and light chains are connected to Conv11 and Conv12 respectively. The extracted features of the heavy and light chains are then concatenated and enter Conv2 for a deeper feature extraction. The final extracted features from Conv2 are then flattened (reduced to one dimension) and concatenated with the additional features (germline, loop lengths and canonical structures) before entering Ff1 and finally into the output nodes. The purpose of Ff1 is to learn each individual residue's role in the paratope based on the extracted features and the additional ones. The architecture is shown in Figure S1. The network was optimized with a focal loss (Lin *et al.*, 2017) and a stochastic gradient descent (SGD) optimizer. The learning rate followed a one-cycle learning rate policy (Smith and Topin, 2017) with a max learning rate of 0.5, a minimum learning rate of 0.1% of the max one and maximum momentum of 0.9. Exponential Linear Units (ELU) were used as activation functions for Conv11, Conv12, Conv2 and Ff1, and sigmoid on the final output. Dropout (Srivastava *et al.*, 2014) and early stopping (Prechelt, 1998) were used throughout training as regularization techniques. All hyperparameters (i.e. nodes, filter sizes, dropout rate etc.) mentioned above were found empirically.

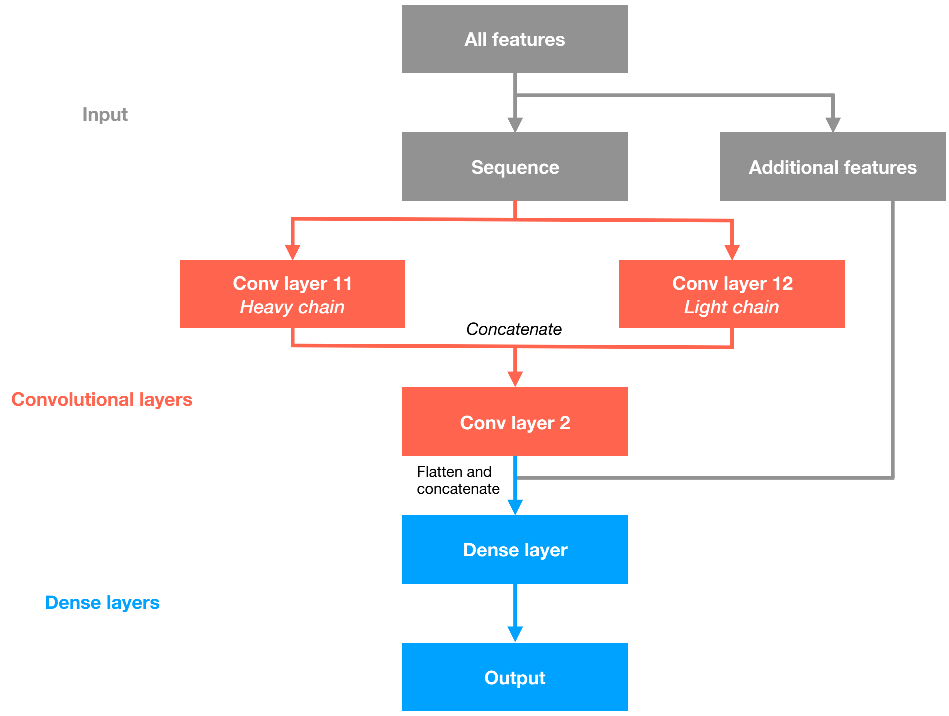

Figure S1: The CNN architecture implemented in the proABC-2 method.

Model evaluation

The evaluation of the model has been performed using 10-fold nested cross validation on the full CNN-set (769 complexes). The performance was measured taking into account three different metrics: area under the Receiver Operating Characteristic curve (AUC), Matthew Correlation Coefficient (MCC) and F-score. MCC and F-score were calculated using a threshold of 0.40, 0.30 and 0.30, respectively for Pt, Hy and Hb.
These cut-offs were selected by averaging the thresholds that for each fold of the cross validation gave the best MCC.

Comparison with Parapred

For a fair comparison proABC-2 was trained on the *Parapred-set* and the AUC, MCC and F-score were calculated on the same residues used by Parapred to make the predictions (CDRs defined according to the Chothia numbering scheme plus two extra residues at both ends) after a 10-fold nested cross validation. For proABC-2 the MCC and F-score were calculated using a threshold of 0.37 (determined as explained in the previous paragraph), while the values from the work of Liberis et al. (Liberis *et al.*, 2018) are reported for Parapred. The results in Table S1 show that proABC-2 outperforms Parapred in terms of AUC and MCC but has a lower performance in terms of F-score.

Table S1: Performance comparison between Parapred and proABC-2

Method	AUC	MCC	F-score
proABC-2	0.91	0.56	0.62
Parapred	0.88	0.55	0.69

Docking scenarios and settings

To assess the impact of the proABC-2 predictions on HADDOCK's docking performance the following docking scenarios were evaluated:

1. *Pred Para – Surf*: No previous information about the epitope is provided to HADDOCK. The docking was performed by using the residues predicted to be in contact by proABC-2, defined as active, and the antigen residues having a relative accessible surface areas (RSA) ≥ 40% calculated with NACCESS (Hubbard SJ, 1993), provided as passive. Default docking settings were used except for the sampling that was increased to 10000, 400, 400 for it0, it1 and water respectively.

2. *Pred Para – Epi 9*: In this case we use a loose definition of the epitope region by selecting all the antigen residues within a 9Å distance from the antibody in the reference structure. We provide to HADDOCK the residues predicted by proABC-2 as active and the defined antigen residues as passive. Default docking settings were used except for the sampling that was increased to 5000, 400, 400 for it0, it1 and water respectively.

The antibody structures were renumbered with an in-house R script using a consecutive numbering as HADDOCK is not able to deal with the insertion format of the Chothia scheme.

Docking evaluation criteria

Docking performance was evaluated by classifying the models into 3 classes: high (***), medium (**) of low (*) quality defined according to the CAPRI criteria (Janin et al., 2003; Méndez et al., 2003) (see Table S2). We calculated the interface root mean square deviation (i-RMSD), the ligand root mean square deviation (L-RMSD) and the fraction of native contacts (F_{nat}) as already reported (Méndez *et al.*, 2003). Briefly, the i-RMSD is calculated on the interface residues backbone atoms defined on the native structure using a 10Å cut-off, the L-RMSD is calculated by superimposing on the antibody backbone atoms and calculating the RMSD on the antigen ones. Finally, F_{nat} is calculated as the number of native contacts in a docking model divided by the total number of contacts in the reference structure. These are defined using a 5Å cut-off.

F_{nat} has been calculated using in-house scripts while fitting and RMSD calculations were performed using the McLachlan algorithm (McLachlan, 1982) as implemented in the program ProFit (http://www.bioinf.org.uk/software/profit/) from the SBGrid distribution (Morin *et al.*, 2013).

Table S2: Classification of docking models in the classes: ***, **, * according to F_{nat}, and either L-RMSD or i-RMSD measures.

Class	F_{nat}	L-RMSD[Å]	i-RMSD[Å]
High (***)	≥ 0.5	≤ 1.0	or ≤ 1.0
Medium (**)	≥ 0.3	≤ 5.0	or ≤ 2.0
Acceptable (*)	≥ 0.1	≤ 10.0	or ≤ 4.0

Impact on docking accuracy

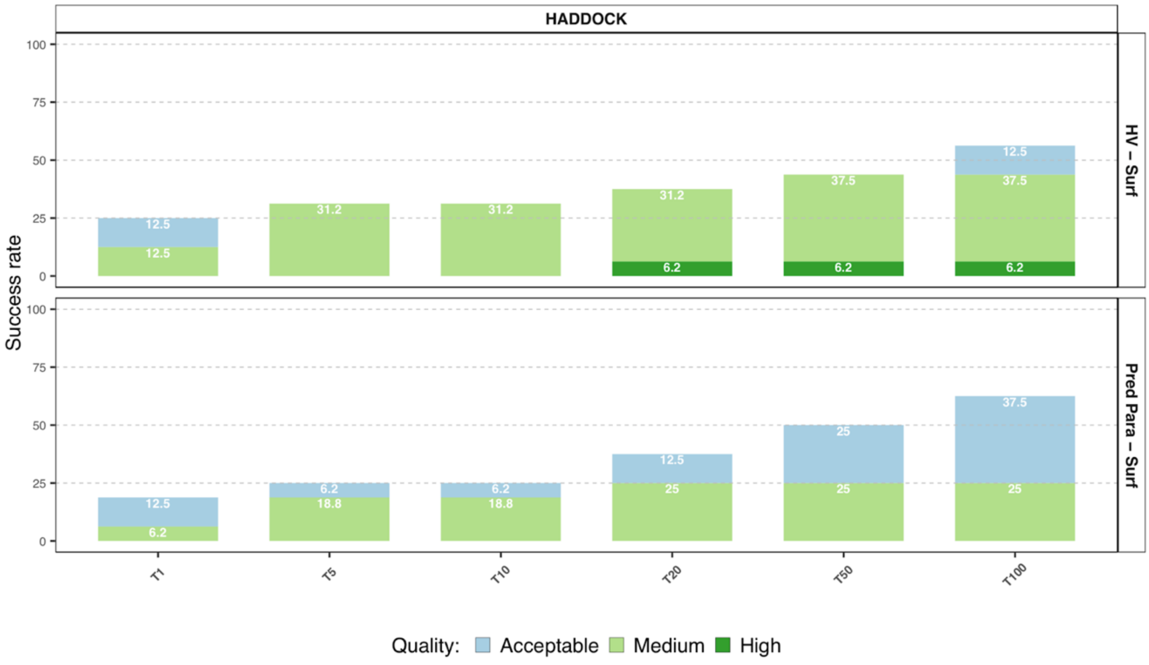

Figure S2: HADDOCK success rate as a function of the top 1, 5, 10, 20, 50 and 100 ranked models. The top row (HV - Surf) shows the success rate using the antibody HV loops and the entire antigen surface as restraints. The second represents the success rate achieved by driving the docking with the proABC-2 predictions and the full surface of the antigen. The colour coding indicates the quality of the models according to CAPRI criteria.

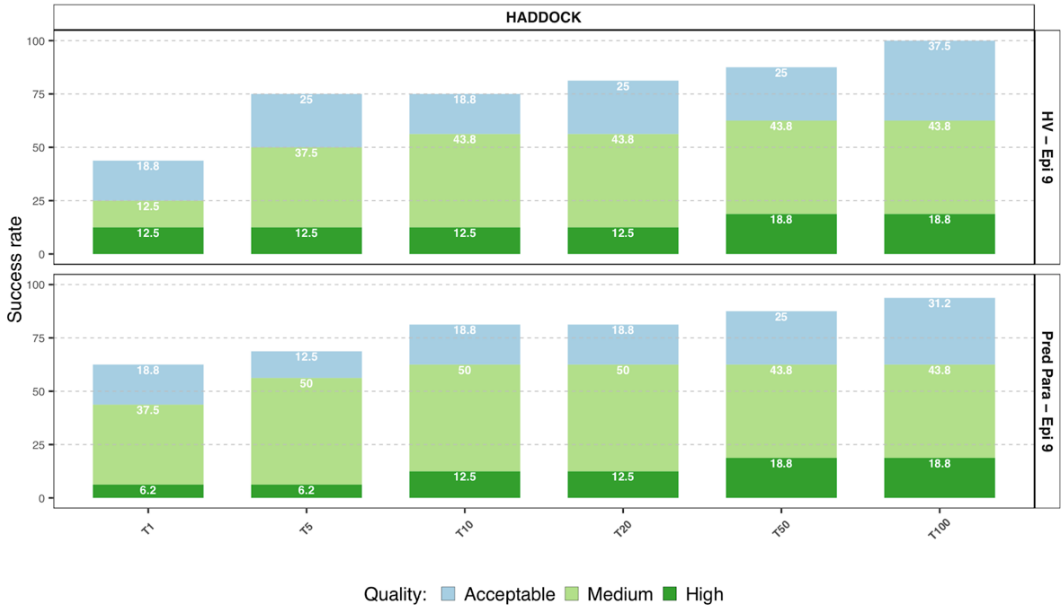

Figure S3*:* HADDOCK success rate as a function of the top 1, 5, 10, 20, 50 and 100 ranked models. The top row (HV – Epi 9) shows the success rate using the antibody HV loops and a loose definition of the epitope using a 9Å cut-off. The second represents the success rate achieved by driving the docking with the proABC-2 predictions and the same definition for the epitope on the antigen. The colour coding indicates the quality of the models according to CAPRI criteria.

Web server

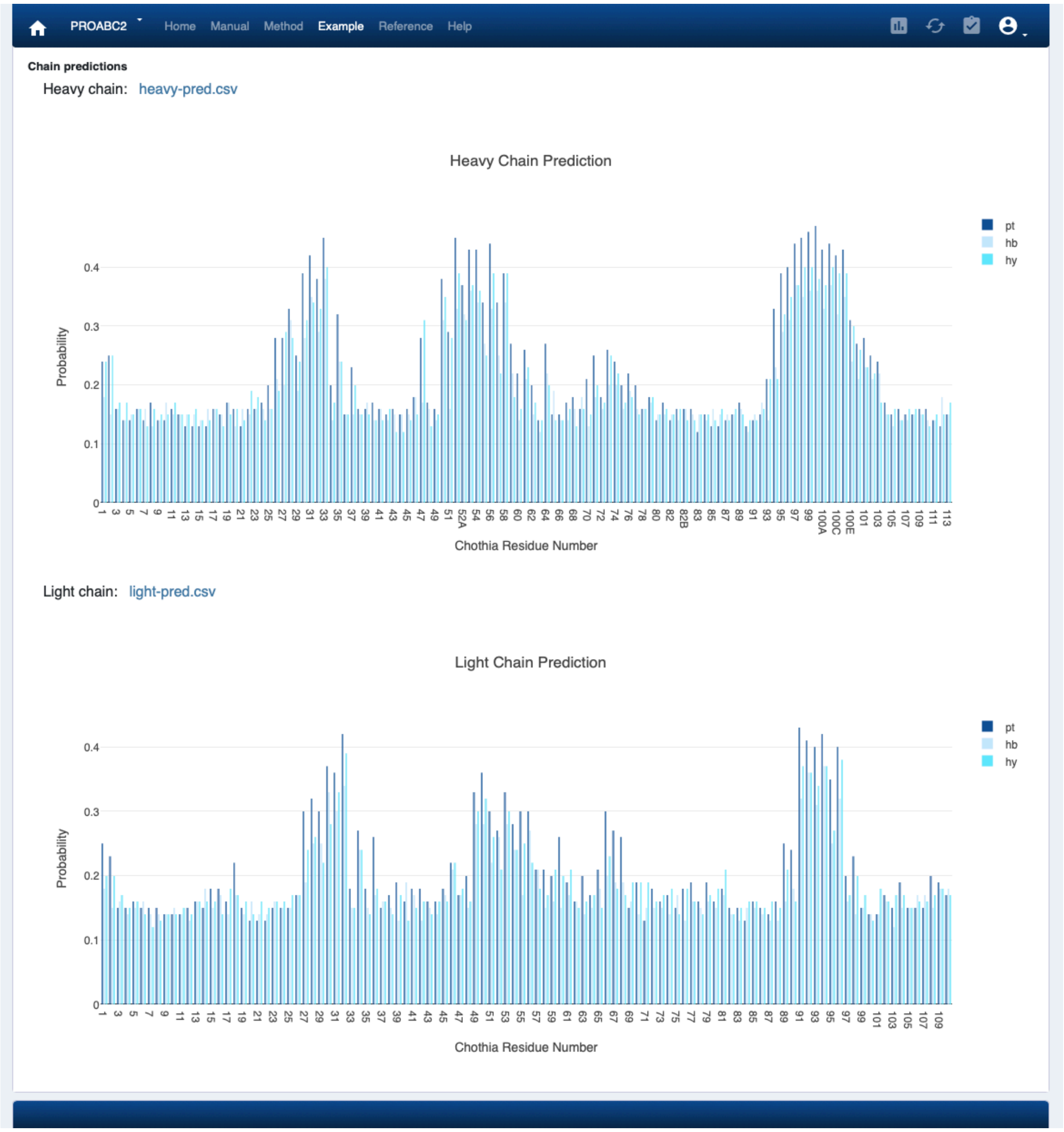

Figure S4: Output page of the proABC-2 web server (https://wenmr.science.uu.nl/proabc2/). It shows the interaction probability of the antibody residues belonging to the heavy and light chain.

References

Abadi,M. *et al.* (2016) TensorFlow: Large-Scale Machine Learning on Heterogeneous Distributed Systems.

Baker,E.N. and Hubbard,R.E. (1984) Hydrogen bonding in globular proteins. *Prog. Biophys. Mol. Biol.*, **44**, 97–179.

Bissantz,C. *et al.* (2010) A Medicinal Chemist's Guide to Molecular Interactions. *J. Med. Chem.*, **53**, 5061–5084.

Chothia,C. and Lesk,A.M. (1987) Canonical structures for the hypervariable regions of immunoglobulins. *J. Mol. Biol.*, **196**, 901–917.

Fu,L. *et al.* (2012) CD-HIT: Accelerated for clustering the next-generation sequencing data. *Bioinformatics*, **28**, 3150–3152.

Hubbard SJ,T.J. (1993) NACCESS. *Comput. Progr.*

Liberis,E. *et al.* (2018) Parapred: Antibody paratope prediction using convolutional and recurrent neural networks. *Bioinformatics*, **34**, 2944–2950.

Lin,T.-Y. *et al.* (2017) Focal Loss for Dense Object Detection.

McLachlan,A.D. (1982) Rapid comparison of protein structures. *Acta Crystallogr. Sect. A*, **38**, 871–873.

Méndez,R. *et al.* (2003) Assessment of blind predictions of protein-protein interactions: Current status of docking methods. *Proteins Struct. Funct. Genet.*, **52**, 51–67.

Morea,V. *et al.* (1998) Conformations of the third hypervariable region in the VH domain of immunoglobulins. *J. Mol. Biol.*, **275**, 269–294.

Morin,A. *et al.* (2013) Collaboration gets the most out of software. *Elife*, **2**.

Prechelt,L. (1998) Early Stopping - But When?, pp. 55–69.

Rodrigues,J. *et al.* (2019) JoaoRodrigues/interfacea: First beta version of the API.

Smith,L.N. and Topin,N. (2017) Super-Convergence: Very Fast Training of Neural Networks Using Large Learning Rates.

Srivastava,N. *et al.* (2014) Dropout: A simple way to prevent neural networks from overfitting. *J. Mach. Learn. Res.*, **15**, 1929–1958.

Vargas-Madrazo,E. and Paz-García,E. (2002) Modifications to canonical structure sequence patterns: Analysis for L1 and L3. *Proteins Struct. Funct. Genet.*, **47**, 250–254.

Vreven,T. *et al.* (2015) Updates to the Integrated Protein-Protein Interaction Benchmarks: Docking Benchmark Version 5 and Affinity Benchmark Version 2. *J. Mol. Biol.*, **427**, 3031–3041.

Ye,J. *et al.* (2013) IgBLAST: an immunoglobulin variable domain sequence analysis tool. *Nucleic Acids Res.*, **41**, W34–W40.
